# Hospital‐associated sarcopenia, acute sarcopenia, and iatrogenic sarcopenia: Prevention of sarcopenia during hospitalization

**DOI:** 10.1002/jgf2.625

**Published:** 2023-04-23

**Authors:** Hidetaka Wakabayashi

**Affiliations:** ^1^ Department of Rehabilitation Medicine Tokyo Women's Medical University Hospital Shinjuku‐ku Tokyo Japan

## Abstract

Sarcopenia can be classified as age‐, activity‐, nutrition‐, and disease‐related. Hospital‐associated sarcopenia, acute sarcopenia, and iatrogenic sarcopenia are activity‐, nutrition‐, and disease‐related, not age‐related. There is considerable overlap between hospital‐associated sarcopenia and acute sarcopenia; however, they are distinct concepts. Some causes of hospital‐associated sarcopenia and acute sarcopenia are iatrogenic.

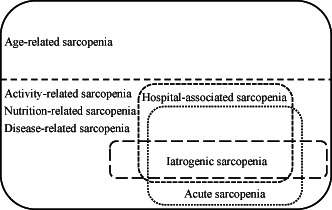

## 1 INTRODUCTION

Sarcopenia is important in primary care because it is a loss of skeletal muscle mass and function that causes bedridden, dysphagia, and respiratory dysfunction. However, the percentage of primary care physicians who are familiar with sarcopenia is quite low at 18.8%.^1^ The causes of sarcopenia can be classified into age, activity, nutrition, and disease. Therefore, sarcopenia can occur in people who are not old due to activity, nutrition, or disease.

**FIGURE 1:**
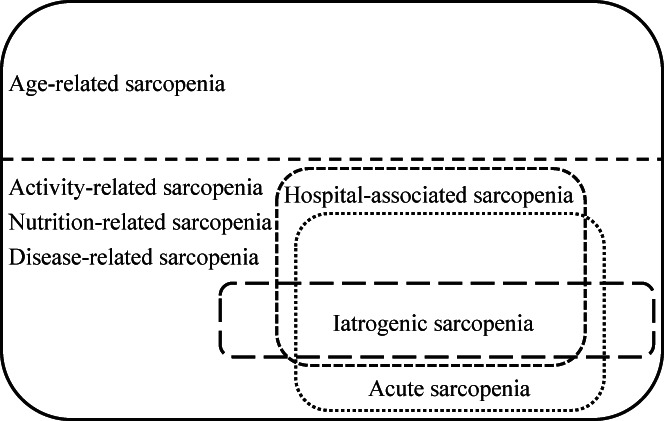
Relationship between hospital‐associated sarcopenia, acute sarcopenia, and iatrogenic sarcopenia. Sarcopenia can be classified as age‐, activity‐, nutrition‐, and disease‐related. Hospital‐associated sarcopenia, acute sarcopenia, and iatrogenic sarcopenia are activity‐, nutrition‐, and disease‐related, not age‐related. There is considerable overlap between hospital‐associated sarcopenia and acute sarcopenia; however, they are distinct concepts. Some causes of hospital‐associated sarcopenia and acute sarcopenia are iatrogenic.

Sarcopenia often occurs during hospitalization in acute care hospitals. Hospital‐associated sarcopenia refers to sarcopenia resulting from hospitalization and is related to hospital‐associated deconditioning and hospital‐associated disability. Hospital‐associated sarcopenia occurs not only in acute care hospitals but also in rehabilitation and long‐term care hospitals. Acute sarcopenia refers to sarcopenia that occurs primarily during an acute hospitalization.^2^ However, acute sarcopenia can occur in institutional and home medical care. Hospital‐associated sarcopenia and acute sarcopenia are distinct concepts, although there is considerable overlap. The causes of hospital‐associated sarcopenia and acute sarcopenia are activity, nutrition, and disease, not age. In addition, the causes of hospital‐associated sarcopenia and acute sarcopenia are classified into non‐iatrogenic and iatrogenic (Figure 1). Iatrogenic sarcopenia refers to sarcopenia caused by the activities of medical staff including doctors, nurses, or other healthcare professionals in healthcare facilities.^3^

### 1.1 Non‐iatrogenic sarcopenia

Activity‐related sarcopenia occurs in bed rest required for medical treatment. For example, if the patient is hemodynamically unstable and sitting causes arrhythmias and dyspnea, bed rest is required.

Nutrition‐related sarcopenia occurs when the patient's food intake is inadequate despite medically appropriate nutritional care management.

Disease‐related sarcopenia occurs with trauma, fractures, cancer, chronic organ failure, and chronic inflammatory diseases and medically necessary surgery.

### 1.2 Iatrogenic sarcopenia

Activity‐related sarcopenia occurs during medically unnecessary bed rest. For example, when the patient is hospitalized for aspiration pneumonia, the physician orders tentative bed rest without appropriate assessment.

Nutrition‐related sarcopenia results from medically inappropriate nutritional care management. For example, nutritional care management is often inadequate in hospitalized patients with aspiration pneumonia who do not take oral nutrition.

Disease‐related sarcopenia occurs with iatrogenic diseases or drug‐related adverse events.

Rehabilitation nutrition^4^ and rehabilitation pharmacotherapy^5^ can be useful in the prevention of hospital‐associated sarcopenia and acute sarcopenia, both non‐iatrogenic and iatrogenic. Rehabilitation nutrition and rehabilitation pharmacotherapy are defined as helping people with disabilities and frail older people to achieve the highest possible body functions, activities, participation, and quality of life (QOL), using holistic evaluation by the International Classification of Functioning, Disability and Health (ICF), rehabilitation nutrition care process, and rehabilitation pharmacotherapy management.

The combination of rehabilitation, appropriate nutritional care management, and medication review from the day of admission can prevent sarcopenia during hospitalization to some extent. Prevention of iatrogenic sarcopenia is possible and should be done at all costs. However, prevention of sarcopenia due to non‐iatrogenic disease is difficult. Primary care physicians working in acute care hospitals should be responsible for managing not only diseases causing hospitalization but also hospital‐associated sarcopenia and acute sarcopenia and should provide rehabilitation nutrition and rehabilitation pharmacotherapy. Clinical practice guidelines for sarcopenia and rehabilitation nutrition are available for primary care physicians. The Global Leadership Initiative in Sarcopenia (GLIS) will develop new consensus papers for sarcopenia.^6^ For prevention of sarcopenia, it is desirable to include hospital‐associated sarcopenia and acute sarcopenia, as well as age‐related sarcopenia in the GLIS.

## CONFLICT OF INTEREST STATEMENT

The author has stated explicitly that there are no conflicts of interest in connection with this article.
